# Cross-continental collaboration for understanding postpartum major depression with psychotic features

**DOI:** 10.3389/fgwh.2022.996501

**Published:** 2022-11-21

**Authors:** Mary Kimmel, Harish Thippeswamy, Astrid Kamperman, H. N. Madhuri, Karen Putnam, Crystal Schiller, Katie Weinel, Hannah Rackers, Janneke Gilden, Veerle Bergink, Samantha Meltzer-Brody, Prabha Chandra

**Affiliations:** ^1^ Department of Psychiatry, University of North Carolina at Chapel Hill School of Medicine, Chapel Hill, NC, United States; ^2^ Department of Psychiatry, National Institute of Mental Health and Neurosciences (NIMHANS), Bangalore, India; ^3^ Department of Psychiatry, Eramus Medical Center, Rotterdam, Netherlands; ^4^ Department of Psychiatry, University of North Carolina at Chapel Hill School of Medicine, Chapel Hill, NC, United States; ^5^ Department of Psychiatry, San Jose Medical Center, San Jose, CA, United States

**Keywords:** postpartum depression, postpartum psychosis, major depression with psychotic features, lithium, ECT

## Abstract

**Purpose:**

Assess postpartum depression and psychotic symptoms from three continents.

**Methods:**

Compare numbers of women with depression and psychotic symptoms, mania with or without psychotic features, or transient non-affective psychosis and medication choice.

**Results:**

The prevalence of postpartum depression and psychosis and treatment choice differed at each site.

**Conclusions:**

Best treatment for postpartum depression with psychotic features has not been established yet. Cross-continental collaboration with similar assessments holds promise to develop best practices for these high risk mother-infant dyads.

## Background

Maternal mood and anxiety disorders including postpartum psychosis (PPP) lead to poor outcomes for mothers and their children and at the worst lead to suicide and infanticide (1, 2). The incidence of first-lifetime onset PPP from population-based register studies of psychiatric inpatient admissions, not including outpatient or individuals that do not get admitted to an inpatient unit, varies from 0.25 to 0.6 per 1,000 births (2). The relative risk for new onset affective psychosis within four weeks of delivery is 23 times higher than any other time in an individual's life; indicating the postpartum period may have unique biologic and psychologic changes that make it a more vulnerable time to develop affective psychosis (2, 3). These prevalence numbers often only consider individuals with no prior history and do not account for individuals with history of bipolar disorder or major depression with psychotic features (MDD w/ PF) that may have a recurrence postpartum. PPP can be considered an umbrella term with the following three distinct subtypes: 1. Mania or mixed episodes (with or without psychotic features) (MM); 2. Depressive episodes with psychotic features (MDD w/ PF); and 3. Acute/transient nonaffective psychotic episodes (AP) (2). There is limited information about the specific group with MDD w/ PF during the perinatal period. Women with depression and psychotic features predominating may be particularly at high risk of completed suicide given the known higher risk outside of the perinatal period (4). From 2017 to 2019 the number one cause of maternal morbidity was death due to mental health including suicide and overdose (5); possibly indicating that PPP is more common than suspected, MDD w/ PF not being optimally treated, and more knowledge is needed about PPP and particularly MDD w/ PF.

Bergink et al. developed an algorithm for treatment of PPP that includes a sequential 4-step treatment algorithm: first benzodiazepines, then antipsychotics, followed by lithium, and then electroconvulsive therapy (ECT) (6). The majority of patients (98.4%) achieved remission prior to the 4th step, but women with depression with psychotic features had a longer duration of episodes compared to the other subgroups. There is a need to investigate effective treatments for women with postpartum depression with psychotic features (6). Recommendation for first line treatments for patients with MDD w/ PF in the general population include antidepressants such as selective serotonin reuptake inhibitors (SSRI) and ECT (7). Previous studies warned against the use of antidepressants in the postpartum period for women with severe acute depression or depression with psychotic features (2, 8). As PPP is highly associated with bipolar disorder, antidepressants have been found to induce mania, rapid cycling and worsen psychosis (6).

Clearly, treatment of women with postpartum depression and psychotic symptoms warrant greater study. Three mother baby units (MBU)/perinatal psychiatry inpatient units (PPIU) from the United States, the Netherlands, and India provide a unique opportunity to study the prevalence and treatment of patients with PPP including the MDD w/ PF subtype.

## Methods

### Study population

Data were collected from the MBU in Bangalore, India (National Institute of Mental Health and Neurosciences or NIMHANS); the MBU in Rotterdam, the Netherlands (Erasmus Medical Center or EMC); and the PPIU in Chapel Hill, North Carolina, United States (University of North Carolina or UNC). Data collection included surveys given to patients and from the medical records of the patients. For the UNC inpatient unit, English-literate pregnant and postpartum women within a year of delivery completed questionnaires including the Edinburgh Postnatal Depression Scale (EPDS) (9) right after admission and before discharge on the UNC inpatient unit as described previously (10, 11). Other data has been collected from the medical record including diagnoses made by the clinical teams and recorded in the medical record. Diagnoses were based on enquiry of the patients current symptoms but also of past history of psychiatric illness and past treatment history and were based on the Diagnostic and Statistical Manual of Mental Disorders (DSM). A detailed description of the NIMHANS MBU set up and the nature of services has been published (12). The data is collected prospectively, from medical records based on standard processes of care for each admitted patient, including all demographics and clinical information regarding obstetrical and mental health history of patient admitted to the MBU within six month of delivery. Participants are fluent in English or Kannada (regional language of state where study was conducted). All of those included are postpartum with illness onset within 6 months of delivery (13). Two qualified psychiatrists diagnose patients through clinical interviews per ICD-10 criteria (13). For the EMC MBU, data was taken from the OPPER-cohort, a prospective study of women with an onset of psychotic and/or manic symptoms within three months postpartum as previously described (6). The goals of data collection are to develop a longitudinal cohort to investigate the biology, prevention, treatment, and long-term outcomes of severe postpartum mood disorders. All provided informed consent to participate in the study. Eligible subjects are women admitted to the MBU at Erasmus Medical Center. The Structured Interview for DSM-IV Axis I Disorders (SCID) (14) was administered during admission to determine diagnosis. The EPDS was administered weekly. Data collection and analysis was approved by the three institutions' Institutional Review Boards/Medical Ethics Committees.

As noted, the UNC diagnoses are based on clinical interviews with the Diagnostic and Statistical Manual of Mental Disorders (DSM) as a guide, NIMHANS diagnoses are based on International Statistical Classification of Diseases and Related Health Problems 10th Revision (ICD-10), and EMC diagnoses are based on the Structured Clinical Interview for DSM. However, before including individuals in the cmbined data set, each site reviewed individual diagnoses from the medical records to ensure all included individuals were categorized according to the subsets of MM, AP, or MDD w/ PF identified by (Kamperman et al. (2017)) (15). Meetings were held between the three sites to ensure consistency with the diagnoses as descrbied by Kamperman. Postpartum subjects must have a primary diagnosis falling within three distinct groups: 1) MM 2) AP 3) MDD w/ PF. Diagnoses of primary psychotic disorders, Schizoaffective Disorder and Schizophrenia, were excluded. A total of 278 women met inclusion criteria (UNC *n* = 29 (August 2011 to March 2016), NIMHANS *n* = 103 (2009 to November 2018), EMC *n* = 146 (August 2005 and January 2015)). These patients were further divided into those with previous psychotic or manic symptoms and those with the onset of psychotic or manic symptoms for the first time during the postpartum period.

The common variables among the three sites included demographic characteristics (age, parity) and outcome measures (diagnosis, EPDS (9) closest to admission and closest to dischrage, length of stay, medications, and whether patient received ECT).

## Results

### Sample characteristics

The mean age of women across the three sites was similar: 30.0 SD. 6.4 (UNC), 26.4 SD. 5.3 (NIMHANS), and 31.2 SD 4.8 (EMC). The majority from UNC and NIMHANS were multiparous (65.5% and 55.3% respectively), but the majority from EMC were primiparous (78.5%). Mean length of stay varied considerably: 9.5 days for UNC, 21.3 days for NIMHANS, and 60.8 days for EMC. About 45% of the women from UNC and similarly 47% from NIMHANS were classified as having first onset psychosis or mania/mixed symptoms. The average EPDS scores at admission were similar (UNC: M = 16.9 (SD = 6.4); NIMHANS: M = 15.8 (SD = 6.1); AMC: M = 13.6 (SD = 6.3)) and then similarly decreased by discharge (8.2 SD. 6.5 for UNC, 7.3 SD. 4.4 for NIMHANS, 4.7 SD. 4.9 for EMC).

### Prevalence

**Figure 1:**
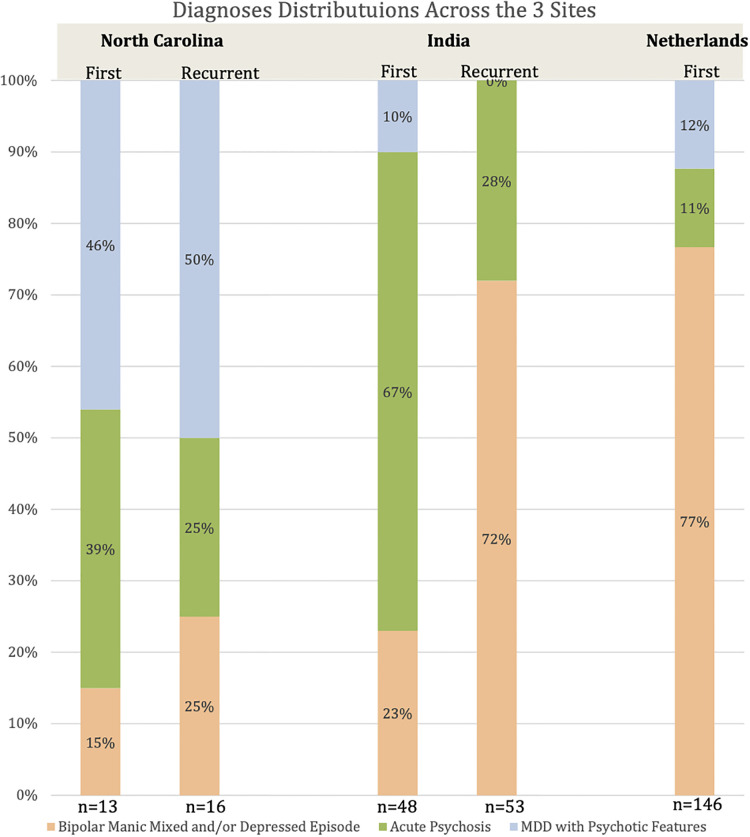
Diagnoses by site with first onset vs. recurrent separated prevalence of each subtype by site and further divided based on whether first onset or recurrent symptoms.

UNC had the highest percent diagnosed with first onset MDD w/ PF (46%) compared to 10% and 12% for NIMHANS and EMC. NIMHANS had the highest percent diagnosed with new onset AP (67%). EMC had the highest percent diagnosed with MM (77%). While NIMHANS had 23% for first onset MM, this increased to 72% for recurrent cases. UNC maintained more similar percentages between the three groups in first onset and recurrent. See Figure 1.

### Treatments

**Figure 2:**
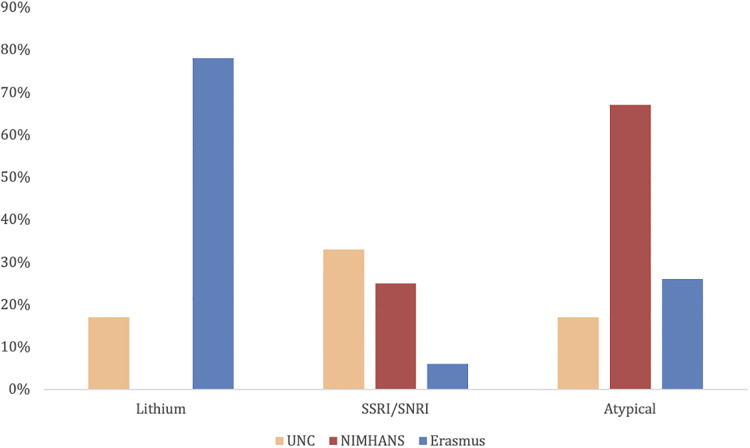
Treatment of first onset postpartum major depressive disorder with psychotic features percentage of patients that received each type of medicine compared by site.

Figure 2 illustrates medications prescribed for those with first onset postpartum MDD w/ PF. EMC was the most likely of the three sites to use lithium compared to other medications and compared to other sites; and while did not treat anyone with MDD w/ PF with Electroconvulsive Therapy (ECT) did treat one individual with MM. NIMHANS did not use lithium and was most likely of the three sites to use an atypical antipsychotic; and was most likely to use ECT with ECT used for three cases of MDD w/ PF, 37 cases of MM and 25 cases of AP. UNC was most likely of the three sites to use an SSRI or SNRI; and only treated one patient with MDD w/ PF with ECT.

## Discussion

This is the first study bringing together data from three specialized psychiatric inpatient units from three continents and the largest subset of those with MDD w/ PF. This has led to two observations: both diagnosis and treatment of postpartum MDD w/ PF in relation to other subtypes of PPP varies across three geographical sites.

Diagnosis of postpartum MDD w/ PF across sites may differ due to multiple considerations: (1) actual differences in numbers of those with predominant depression and psychotic symptoms; (2) provider differences in diagnosis despite using the DSM and ICD-10 (e.g., cultural differences in stigma and acceptability of the three subtypes); and (3) cultural impacts on what information patients share with providers. A longitudinal study could determine if those with first onset psychosis from NIMHANS would later be diagnosed with mania or a mixed episode. Our findings may indicate biases in the US against identifying manic symptoms, but also shorter length of stay may contribute to less ability to ascertain and observe cycling of mood and mixed states. Another contributing factor may be that the cohorts differed in average amount of time from delivery with EMC patients being closest to delivery and UNC patients being furthest from delivery. Larger longitudinal studies would improve the ability to study greater number of factors including trajectories of symptoms, time from delivery, and cultural factors.

Overall diagnostic practices per site seem to correlate with variation in medication choice for first onset MDD w/ PF at each site. While there is evidence that ECT is more effective in the postpartum period than other times and ECT works more quickly than medication (16), its use was variable across sites. The largest factor complicating the use of lithium is its use in relation to breastfeeding. There is consensus that data about its impact on the infant is limited but this leads to some recommending not supporting breastfeeding with lithium use while others suggest it is not contraindicated with a clear risk/benefit discussion (17, 18). Antidepressants are more compatible with breastfeeding and more acceptable than ECT but hold the possibility to worsen course for some women and the effectiveness of antidepressants for MDD w/ PF is less clear (8). Greater knowledge of symptom trajectories could improve ability to personalize medication regimens and determine if use of a medication such as lithium is particularly important for an individual patient.

It is critical to assess for psychotic and mixed/manic symptoms in addition to depression and anxiety symptoms. Increased sample size is required to integrate molecular factors and social factors with more robust combinations of symptoms including psychotic and mixed/manic symptoms. Cross-cultural studies will further improve the ability to ensure psychotic and mixed/manic symptoms can be assessed through the lens of cultural impacts. The studies from these high risk women in specialized units could then inform identification and treatment in outpatient psychiatric and primary care settings.

## Conclusion

This cross-continental study highlights the differences in prevalence and treatment making it difficult to recommend standard practices for the group of patients with depression and psychotic symptoms in the postpartum period. ECT and lithium seem good treatment options but more case series are needed. Future collaboration to study biologic and social contributors would improve understanding of how a presentation with depression and psychotic symptoms may differ and what is the best treatment.

Conflicts of Interest: While not directly related to this paper, the UNC Perinatal Psychiatry Inpatient Unit has received funds from Sage Therapeutics to carry out clinical trials and other research. MCK and SMB have received royalties from UpToDate for two articles about psychotropic medications in relation to lactation.

## Data Availability

The original contributions presented in the study are included in the article/Supplementary Material, further inquiries can be directed to the corresponding author/s.
